# A systematic review identifying common data items in neonatal trials and assessing their completeness in routinely recorded United Kingdom national neonatal data

**DOI:** 10.1186/s13063-019-3849-7

**Published:** 2019-12-16

**Authors:** Sena Jawad, Neena Modi, A. Toby Prevost, Chris Gale

**Affiliations:** 1 Neonatal Medicine, School of Public Health, Faculty of Medicine, Imperial College London, Chelsea and Westminster Hospital Campus, London, SW10 9NH UK; 2 Imperial Clinical Trials Unit, School of Public Health, Imperial College London, London, W12 7RH UK

**Keywords:** Common data items, Data quality, NNRD, Efficient trials, Electronic patient records, Electronic health records, Neonatal clinical trials

## Abstract

**Background:**

We aimed to test whether a common set of key data items reported across high-impact neonatal clinical trials could be identified, and to quantify their completeness in routinely recorded United Kingdom neonatal data held in the National Neonatal Research Database (NNRD).

**Methods:**

We systematically reviewed neonatal clinical trials published in four high-impact medical journals over 10 years (2006–2015) and extracted baseline characteristics, stratification items and potential confounders used to adjust primary outcomes. Completeness was examined using data held in the NNRD for identified data items, for infants admitted to neonatal units in 2015. The NNRD is a repository of routinely recorded data extracted from neonatal Electronic Patient Records (EPR) of all admissions to National Health Service (NHS) Neonatal Units in England, Wales and Scotland. We defined missing data as an empty field or an implausible value. We reported common data items as frequencies and percentages alongside percentages of completeness.

**Results:**

We identified 44 studies involving 32,095 infants and 126 data items. Fourteen data items were reported by more than 20% of studies. Gestational age (95%), sex (93%) and birth weight (91%) were the most common baseline data items. The completeness of data in the NNRD was high for these data with greater than 90% completeness found for 9 of the 14 most common items.

**Conclusion:**

High-impact neonatal clinical trials share common data items. In the United Kingdom, these items can be obtained at a high level of completeness from routinely recorded data held in the NNRD. The feasibility and efficiency using routinely recorded EPR data, such as that held in the NNRD, for clinical trials, rather than collecting these items anew, should be examined.

**Trial registration:**

PROSPERO registration number CRD42016046138. Registered prospectively on 17 August 2016.

## Introduction

High-quality randomised controlled trials are considered the gold standard research approach to identify causality or demonstrate treatment efficacy. There are many treatment uncertainties in neonatal practice [1] that would benefit from being subjected to high-quality randomised clinical trials [2]. However, the high cost of undertaking large and methodologically robust trials [3] means that only a small number are undertaken each year: the median cost of randomised controlled trials was estimated between US$43 and US$103,254 per participant [4] and publicly funded pragmatic neonatal trials cost £1.5–2 million [5]. A key driver of cost in clinical trials is data collection; the mean costs of trial data collection using conventional Case Record Forms have been estimated to be €1135 per participant [6]. More efficient collection; for example, using electronic Case Record Forms [6] and routinely available clinical data [7], provide opportunities to reduce costs and facilitate neonatal trials to improve the limited evidence base upon which much of neonatal care currently relies.

Methods to increase the efficiency of clinical trial data collection have been described by organisations such as the Institute of Medicine [8] and the Clinical Trials Transformation Initiative [9]; these include a targeted collection of common core data items, and extraction of trial data from existing sources, such as Electronic Patient Record (EPR) systems or disease registries; these approaches are most likely to be applicable to pragmatic trials [10]. The use of existing ‘real-world’ data sources such as these provides additional advantages: they can provide up-to-date incidence estimates for baseline and outcome event rates to better inform sample size calculations, and the accuracy and completeness of key data items can be estimated in advance from historical data to inform trial feasibility at the planning stage, and address widely held concerns about poor quality of data from existing sources [11]. However, because not all data items held within a routinely recorded database or registry will be relevant to clinical trials, the data items that are ‘core’ [9] for clinical trials in a particular clinical area need to be established. Established approaches exist for the definition of Core Outcome Sets [12], but none for core *non-outcome* data for clinical trials; for example, baseline or background data, and items used in randomisation.

An increasing proportion of neonatal Cochrane reviews are inconclusive because of insufficient high-quality data from randomised trials [2]. Neonatal care in the United Kingdom is well placed to develop large, efficient trials that use existing data: all infants admitted for National Health Service (NHS) neonatal care in England, Scotland and Wales have clinical data recorded in a summary EPR system as part of routine clinical care, and predefined data [13] are extracted to form the National Neonatal Research Database (NNRD). The effectiveness and efficiency of using routinely recorded clinical data, held in the NNRD for data-enabled neonatal trials, are currently being investigated [14]. We hypothesised that a set of common data items have been reported across neonatal trials that impact clinical practice; the aim of this study was to identify common neonatal data items. As there is no established approach for the identification of common baseline data items we undertook a systematic review to identify baseline data items reported in neonatal trials. A secondary aim was to quantify the completeness of these commonly reported items in the NNRD to inform whether this could be used as the sole or principal data source for clinical trials.

## Methods

### Systematic review

To identify data commonly reported in neonatal trials we conducted a systematic review of neonatal clinical trials published in high-impact journals. We developed a protocol with explicitly defined objectives, information to be extracted, and statistical methods. We prospectively registered the protocol with PROSPERO International Prospective Register of Systematic Reviews, registration number CRD42016046138 (https://www.crd.york.ac.uk/prospero), registered on 17 August 2016.

We searched the four most highly cited general medical journals that publish neonatal trials [15] (*New England Journal of Medicine*, *Lancet*, *British Medical Journal* and *Journal of the American Medical Association*) over a 10-year period from 1 January 2006 to 31 December 2015, using the PubMed database. The PubMed search strategy is described in Additional file 1. We extracted randomised clinical trials written in English that tested an intervention delivered to newborn infants in a neonatal unit setting, with no restriction on the disease area or treatment type. Prior to data extraction we changed the inclusion criteria for studies to include trials of infants born at more than 34 gestational weeks, so that the results would be more generalisable to neonatal trials. We did not include trials where an intervention was applied to a pregnant mother and infant outcomes were reported. Two authors (SJ and CG) independently performed the screening of each potentially relevant record and reviewed full text where necessary to assess eligibility. Discrepancies between the authors were resolved through discussion.

Two authors (SJ, CG) independently extracted the following items from included clinical trials: baseline items, items used in stratification or minimisation (randomisation), and items used to adjust primary outcomes. Other study characteristics that we extracted included whether the trial was multicentre and whether it involved preterm or term infants. Outcome data were not extracted as these are the subject of other parallel work [16]. A comprehensive list of reported data items and frequencies was extracted. Items were combined where appropriate; for example, administration of different medications was combined into the item ‘medications’. Preterm studies were defined as studies involving babies with a gestational age of less than 37 weeks or weighing less than 1500 g and term studies as studies on babies born at or above 37 weeks’ gestation. A formal risk of bias assessment was not conducted as the interest of this study was limited to the data collected, not the interventions or the measure of efficacy.

### Data completeness

Data completeness in the NNRD was examined for infants born in England, Scotland and Wales during the period 1 January 1 2015 to 31 December 2015 for the first seven postnatal days. The NNRD contains over 400 different data per each baby; data held in the NNRD are extracted from individual infants’ EPR data routinely recorded by healthcare professionals as part of clinical care. Details of the Neonatal Dataset are searchable at the following webpage [13] and descriptive data for infants within the NNRD are available here [17]. We calculated the completeness in the NNRD of each data item reported by at least 20% of clinical trials included in the systematic review.

We defined incompleteness as an empty field or an implausible value. Where an item identified through the systematic review (for example, *birth weight*) directly matched a corresponding NNRD field, the completeness of these items was directly calculated. Where an item identified in the systematic review mapped to several fields in the NNRD (for example, *respiratory support*, identified in the systematic review, maps to several NNRD fields, including use of respiratory support, mode of ventilation, non-invasive respiratory support, nitric oxide, tracheostomy, surfactant [13], completeness was determined by at least one value that was not missing or implausible (according to the neonatal dataset data dictionary definition) over the multiple possible NNRD fields.

## Results

### Systematic review

**Fig. 1:**
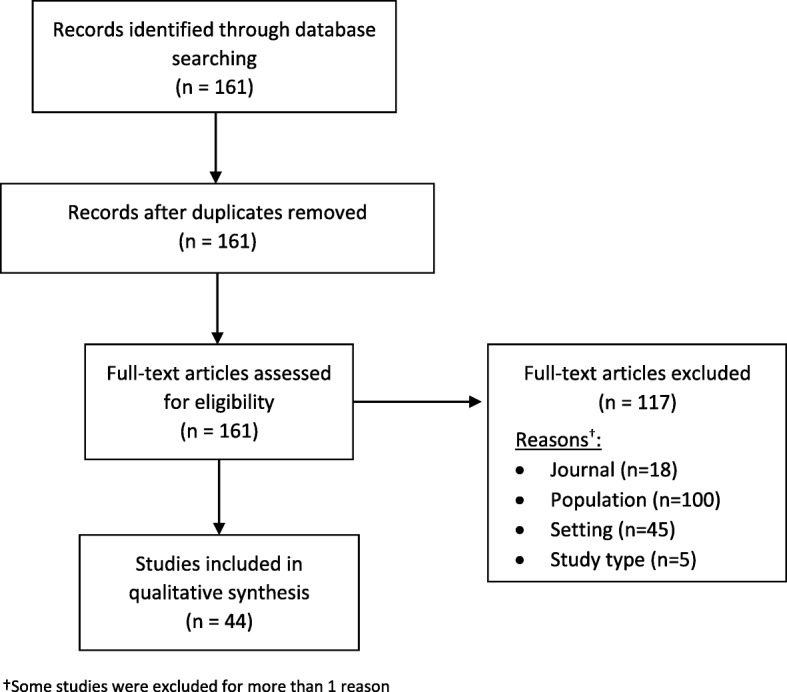
Flow of studies through the systematic review

We identified 161 articles in the literature search. We excluded 117 articles leaving 44 eligible to be included in the review (Fig. 1). Twenty-nine studies included only preterm babies, six only term babies and nine studies included both term and preterm babies (Table 1). The majority of studies (91%) were multicentre trials and overall included 30,968 participants (Table 1).

**Table 1:** The identified studies and their characteristics

Author and year	Title	*N*^a^	Intervention arm	Comparator arm	Single/multiple centre trial	Age/ weight Inclusion criteria of participants	Infant age group	Disease area
Azzopardi 2009 [18]	Moderate hypothermia to treat perinatal asphyxial encephalopathy	325	Total body cooling and intensive care	Intensive care	Multiple	≥ 36 weeks’ gestation	Term	Neurological
Azzopardi 2014 [19]	Effects of hypothermia for perinatal asphyxia on childhood outcomes	325	Standard care with hypothermia	Standard care	Multiple	≥ 36 weeks	Term	Neurological
Ballard 2006 [20]	Inhaled nitric oxide in preterm infants undergoing mechanical ventilation	582	Nitric oxide	Placebo	Multiple	< 32 weeks	Preterm	Respiratory
Bassler 2015 [21]	Early inhaled budesonide for the prevention of bronchopulmonary dysplasia	856	Early inhaled budesonide	Placebo	Multiple	23^+ 0^ to 27^+ 6^ weeks^+days^	Preterm	Respiratory
Baud 2016 [22]	Effect of early low-dose hydrocortisone on survival without bronchopulmonary dysplasia in extremely preterm infants (PREMILOC): a double-blind, placebo-controlled, multicentre randomised trial	521	Hydrocortisone	Placebo	Multiple	24^+ 0^ to 27^+ 6^ weeks^+days^	Term	Respiratory
Beardsall 2008 [23]	Early insulin therapy in very-low-birth-weight infants	386	Early insulin	Standard neonatal care	Multiple	< 1500 g	Preterm	Other- metabolic/endocrine
Benjamin 2014 [24]	Effect of fluconazole prophylaxis on candidiasis and mortality in premature infants, a randomized clinical trial	361	Fluconazole	Placebo	Multiple	< 750 g	Preterm	Infection
Brocklehurst 2011 [25]	Treatment of neonatal sepsis with intravenously administered immune globulin	3493	Polyvalent IgG immune globulin	Placebo	Multiple	< 1500 g	Preterm	Infection
Carlo 2010 [26]	Target ranges of oxygen saturation in extremely preterm infants	1316	Oxygen saturation 85–89%	Oxygen saturation 91–95%	Multiple	24^+ 0^ to 27^+ 6^ weeks^+days^	Preterm	Respiratory
Carr 2009 [27]	Granulocyte-macrophage colony stimulating factor administered as prophylaxis for reduction of sepsis in extremely preterm, small-for-gestational age neonates (PROGRAMS): a single-blind, multicentre randomised controlled trial	280	Granulocyte-macrophage colony stimulating factor	Standard care	Multiple	≤ 31 weeks	Preterm	Infection
Ceelie 2013 [28]	Effect of intravenously administered paracetamol on postoperative morphine requirements in neonates and infants undergoing major noncardiac surgery	71	Continuous morphine	Intermittent intravenously administered paracetamol	Single	> 36^+ 1^ week^+days^ to 1 year	Term	Other- pain
Costeloe 2016 [29]	*Bifidobacterium breve* BBG-001 in very preterm infants: a randomised controlled phase 3 trial	1310	Probiotic *B breve* BBG-001	Placebo	Multiple	23^+ 0^ to 30^+ 6^ weeks^+days^	Preterm	Infection
Davidson 2016 [30]	Neurodevelopmental outcome at 2 years of age after general anaesthesia and awake-regional anaesthesia in infancy (GAS): an international multicentre, randomised controlled trial	719	Awake-regional anaesthesia	General anaesthesia	Multiple	≥ 26 weeks to 60 weeks	Both	Other- sedation/anaesthesia
Fergusson 2012 [31]	Effect of fresh red blood cell transfusions on clinical outcomes in premature, very-low-birth-weight infants	377	Fresh red blood cell transfusions	Standard red blood cell transfusions	Multiple	< 1250 g	Preterm	Other- haematological
Finer 2010 [32]	Early continuous positive airway pressure (CPAP) versus surfactant in extremely preterm infants	1316	Intubation and surfactant	Continuous positive airway pressure	Multiple	24^+ 0^ to 27^+ 6^ weeks^+days^	Preterm	Respiratory
Fivez 2016 [33]	Early versus late parenteral nutrition in critically ill children	1440	Late parenteral nutrition	Early parenteral nutrition	Multiple	Term newborns to 17 years	Term	Other- nutrition
Gopel 2011 [34]	Avoidance of mechanical ventilation by surfactant treatment of spontaneously breathing preterm infants: an open-label randomised, controlled trial	220	Surfactant without ventilation	Standard care	Multiple	26 to 28^+ 6^ weeks^+days^	Preterm	Respiratory
Harris 2013 [35]	Dextrose gel for neonatal hypoglycaemia (the Sugar Babies study): a randomised, double-blind, placebo-controlled trial	237	Dextrose gel	Placebo	Single	35 to 42 weeks	Both	Other- metabolic/endocrine
Hyttel-Sorenson 2015 [36]	Cerebral near infrared spectroscopy oximetry in extremely preterm infants: phase II randomised clinical trial	166	Cerebral near infrared spectroscopy monitoring	Blinded near infrared spectroscopy monitoring	Multiple	< 27^+ 6^ weeks^+days^	Preterm	Neurological
Kelleher 2013 [37]	Oronasopharyngeal suction versus wiping of the mouth and nose at birth: a randomised equivalency trial	488	Gentle wiping of the face, mouth and nose with a towel	Suction with a bulb syringe of the mouth and nostrils	Single	≥ 35 weeks	Both	Respiratory
Kimberlin 2011 [38]	Orally administered acyclovir suppression and neurodevelopment after neonatal herpes	74	Oral acyclovir	Placebo	Multiple	> 800 g	Both	Infection
Kimberlin 2015 [39]	Valganciclovir for symptomatic congenital cytomegalovirus disease	96	Valganciclovir therapy	Placebo	Multiple	≥ 32 weeks	Both	Infection
Kirpalani 2013 [40]	A trial comparing non-invasive ventilation strategies in preterm infants	1007	Nasal intermittent positive-pressure ventilation	Nasal continuous positive airway pressure	Multiple	< 30 weeks and < 1000 g	Preterm	Respiratory
Leuchter 2014 [41]	Association between early administration of high-dose erythropoietin in preterm infants and brain magnetic resonance imaging (MRI) abnormality at term-equivalent age	165	Recombinant human erythropoietin	Placebo	Multiple	26 weeks to 31^+ 6^ weeks^+days^	Preterm	Neurological
Makrides 2009 [42]	Neurodevelopmental outcomes of preterm infants fed high-dose docosahexaenoic acid	657	High docosahexaenoic acid diet	Standard docosahexaenoic acid diet	Multiple	< 33 weeks	Preterm	Other- nutrition
Manley 2013 [43]	High-flow nasal cannulae in very preterm infants after extubation	303	High-flow nasal cannulae	Nasal continuous positive airway pressure	Multiple	< 32 weeks	Preterm	Respiratory
Manzoni 2007 [44]	A multicentre, randomized trial of prophylactic fluconazole in preterm neonates	322	Fluconazole	Placebo	Multiple	< 1500 g	Preterm	Infection
Manzoni 2009 [45]	Bovine lactoferrin supplementation for prevention of late-onset sepsis in very-low-birth-weight neonates	472	Lactoferrin	Lactoferrin + *Lactobacillus rhamnosus* GG Placebo	Multiple	< 1500 g	Preterm	Infection
Mercier 2010 [46]	Inhaled nitric oxide for prevention of bronchopulmonary dysplasia in premature babies (EUNO): a randomised controlled trial	800	Inhaled nitric oxide	Placebo	Multiple	24^+ 0^ to 28^+ 6^ weeks^+days^	Preterm	Respiratory
Morley 2008 [47]	Nasal CPAP or intubation at birth for very preterm infants	610	CPAP	Intubation and ventilation at 5 min	Multiple	25^+ 0^ to 28^+ 6^ weeks^+days^	Preterm	Respiratory
Morris 2008 [48]	Aggressive versus conservative phototherapy for infants with extremely low birth weight	1974	Aggressive phototherapy	Conservative phototherapy	Multiple	501–1000 g	Preterm	Other- hepatic
Morris 2013 [49]	Percutaneous vesicoamniotic shunting versus conservative management for fetal lower urinary tract obstruction (PLUTO): a randomised trial	31	Percutaneous vesicoamniotic shunting	Conservative management	Multiple	No age or weight criteria	Both	Genitourinary
Moss 2006 [50]	Laparotomy versus peritoneal drainage for necrotising enterocolitis (NEC) and perforation	117	Primary peritoneal drainage	Laparotomy with bowel resection	Multiple	< 34 weeks, < 1500 g	Preterm	Gastrointestinal
Natalucci 2016 [51]	Effect of early prophylactic high-dose recombinant human erythropoietin in very preterm infants on neurodevelopmental outcome at 2 years	365	Prophylactic early high-dose recombinant human erythropoietin (rhEPO)	Placebo	Multiple	26^+ 0^ to 31^+ 6^ weeks^+days^	Preterm	Neurological
Schmidt 2012 [52]	Survival without disability to age 5 years after neonatal caffeine therapy for apnea of prematurity	1640	Caffeine therapy	Placebo	Multiple	500–1250 g	Preterm	Respiratory
Schmidt 2013 [53]	Effects of targeting higher versus lower arterial oxygen saturations on death or disability in extremely preterm infants	1201	Oxygen saturation 85–89%	Oxygen saturation 91–95%	Multiple	23^+ 0^ to 27^+ 6^ weeks^+days^	Preterm	Respiratory
Shankaran 2012 [54]	Childhood outcomes after hypothermia for neonatal encephalopathy	190	Hypothermia	Usual care	Multiple	≥ 36 weeks	Both	Neurological
Shankaran 2014 [55]	Effect of depth and duration of cooling on deaths in the neonatal intensive care unit (NICU) among neonates with hypoxic ischemic encephalopathy, a randomised clinical trial	364	32 °C for 72 h 33.5 °C for 120 h 32 °C for 120 h	33.5 °C for 72 h	Multiple	≥ 36 weeks	Both	Neurological
Slater 2010 [56]	Orally administered sucrose as an analgesic drug for procedural pain in newborn infants: a randomised controlled trial	44	Sucrose solution	Sterile water	Single	37–43 weeks	Term	Other- pain
Stenson 2013 [57]	Oxygen saturation and outcomes in preterm infants	2448	Oxygen saturation of 85–89%	Oxygen saturation of 91–95%	Multiple	< 28 weeks	Preterm	Respiratory
Taddio 2006 [58]	Intravenously administered morphine and topically administered tetracaine for treatment of pain in preterm neonates undergoing central-line placement	132	Tetracaine or morphine or both	Neither tetracaine nor morphine	Multiple	No age or weight criteria	Both	Other- pain
Tarnow-Mordi 2016 [59]	Outcomes of two trials of oxygen-saturation targets in preterm infants	1858	Lower oxygen-saturation range	Higher oxygen-saturation range	Multiple	< 28 weeks	Preterm	Respiratory
Vaucher 2012 [60]	Neurodevelopmental outcomes in the early CPAP and pulse oximetry trial	990	Early CPAP with a limited ventilation strategy	Early surfactant administration (2 × 2 factorial) Also to: 85–89% oxygen saturation or 91–95% oxygen saturation	Multiple	24^+ 0^ to 27^+ 6^ weeks^+days^	Preterm	Respiratory
Zivanovic 2014 [61]	Late outcomes of a randomized trial of high-frequency oscillation in neonates	319	High-frequency oscillatory ventilation	Conventional ventilation	Multiple	< 29 weeks	Preterm	Respiratory

^a^Number of infants presenting baseline characteristics

The median number of baseline data items reported in the 44 included trials was 12. Gestational age, sex and birth weight were collected as baseline items for 42 of 44 studies (Table 2). Fourteen data items were reported by at least 20% of studies; 66 baseline data items were reported by one study alone (Additional file 2: Table S1). No study reported all 14 of the most common data items.

**Table 2:** Data items reported in more than 20% of studies and stratified by the age of the study participants

	Infant age							
	Preterm studies (*n* = 29)		Term studies (*n* = 6)		Mixed-ages studies (*n* = 9)		All studies (*n* = 44)	
Baseline Characteristics								
Gestational age	29	(100%)	4	(67%)	9	(100%)	42	(96%)
Sex	29	(100%)	6	(100%)	6	(67%)	41	(93%)
Birth weight	29	(100%)	5	(83%)	6	(67%)	40	(91%)
Antenatal steroids	25	(86%)	1	(17%)	1	(11%)	27	(61%)
Multiple births	21	(72%)	1	(17%)	2	(22%)	24	(55%)
Respiratory support	17	(59%)	3	(50%)	3	(33%)	23	(52%)
Mode of delivery	14	(48%)	2	(33%)	5	(56%)	21	(48%)
Infection	15	(52%)	3	(50%)	3	(33%)	21	(48%)
Drug treatment	15	(52%)	0	(0%)	5	(56%)	20	(45%)
Maternal ethnicity	15	(52%)	1	(17%)	3	(33%)	19	(43%)
Apgar score 5 min	14	(48%)	0	(0%)	5	(56%)	19	(43%)
Age	11	(38%)	6	(100%)	2	(22%)	19	(43%)
Inborn	13	(45%)	0	(0%)	2	(22%)	15	(34%)
Maternal age	6	(21%)	1	(17%)	6	(67%)	13	(30%)
Stratification items								
Neonatal unit identifier	22	(76%)	1	(17%)	2	(22%)	25	(57%)
Gestational age	14	(48%)	1	(17%)	3	(33%)	17	(39%)
Primary outcome adjusting items								
Gestational age	17	(59%)	1	(8%)	1	(11%)	19	(43%)
Neonatal unit identifier	10	(34%)	1	(8%)	2	(22%)	13	(28%)
Birth weight	9	(31%)	0	(0%)	1	(11%)	10	(22%)

Sixteen stratification items were reported by 35 trials. Neonatal unit identifier (57%) and gestational age (39%) were the most common items used for stratification during randomisation. Two (13%) of these stratification items were reported by more than 20% of trials and 9 (56%) were reported by one study only (Additional tables). Twenty-four items were reported by 33 trials to adjust the primary outcome. Of these, 3 (13%) were reported by more than 20% of all trials and 12 (50%) were reported by one study only (Additional file 2 Tables S1, S2, S3, S4). Eight (50%) stratification and 9 (38%) adjustment items were in the top 14 background data items. A full list of all common items can be found in the Additional file 2 Tables S1, S2, S3, S4.

### Data completeness

In 2015, 96,699 infants were admitted to 180 neonatal units in England, Wales and Scotland. Admitted infants received 472,187 days of neonatal care during the first 7 days following birth (data not shown).

The completeness of common data items in the NNRD are summarised by age groups in Table 3. Data completeness in the NNRD is 99.9% for gestational age at birth, 99.9% for sex, 100% for birth weight, 99.7% for multiple birth and 100% for respiratory support on day 1 (Table 3). The majority of data items were more than 90% complete, exceptions include maternal ethnicity (70.2%), mode of delivery (81.4%) and Apgar score at 5 min (79.1%). Completeness was higher for all data items for preterm (mean completeness 94.4%) compared to term babies (mean completeness 89.2%) (Table 3).

**Table 3:** Data completeness in the National Neonatal Research Database (NNRD) for the data items reported in 20% of studies or more

	Age			
	Preterm (*n* = 37,424) (%)	Term (*n* = 59,130) (%)	Unknown (*n* = 145) (%)	All (*n* = 96,699) (%)
Gestational age	100.0	100.0	0	99.9
Sex	99.9	99.9	99.3	99.9
Birth weight	100.0	100.0	91.7	100.0
Antenatal steroids	94.5	89.7	4.8	91.4
Maternal ethnicity	75.6	66.9	1.4	70.2
Multiple births	100.0	99.8	11.7	99.7
Mode of delivery	90.7	75.7	2.8	81.4
Apgar score at 5 min	87.6	73.9	0.7	79.1
Maternal age	96.6	89.2	3.4	92.0
Inborn^a^	98.8	96.6	6.2	97.3
Drug treatment in the first 1 day^bc^				91.9
Respiratory support in the first 1 day^c^				100.0

^a^Corresponding NNRD data item: place of birth

^b^Corresponding NNRD data item: any medication recorded on day 1 of admission

^c^For babies less than 28 weeks gestational age (*n* = 1967)

## Discussion

We have identified a common set of non-outcome data items reported in high-impact neonatal trials. We find that 12 of these 14 data items can be obtained from the NNRD with high completeness for most items (Table 3). The common data items identified here have previously been validated against independently collected trial data [17] where they were shown to be highly accurate and complete in the NNRD. This supports the assertion that non-outcome data held in the NNRD can be used to support large, efficient neonatal trials. We recognise that the trials included in the systematic review also reported a wide range of additional non-outcome data items that were not included in the common set identified here. In planning future pragmatic neonatal trials, the completeness and accuracy of additional data items critical to the integrity of a planned trial can be evaluated using approaches similar to those applied here. However, the finding that reported data items were variable even between similar trials (Additional file 2: Table S2) suggests that some reported data items may not have been critical to trial integrity, and that harmonisation of non-outcome data items may improve the consistency and efficiency of future neonatal trials. The common non-outcome data items we identify here, and their completeness and accuracy [17] in the NNRD, can be used to assess the suitability and feasibility of using the NNRD and other similar routinely recorded data sources for neonatal trials.

Data completeness of the NNRD has previously been calculated by Battersby et al. [17] in relation to a single clinical trial between 2008 and 2015. In this study percentage completeness was very similar to that found in the present study where common data items examined multiple births, gestational age, sex and birth weight, indicating that data completeness within the NNRD for these items is consistent over time. The present study builds upon this work by examining completeness for a wider range of empirically identified non-outcome data items; therefore, extending the relevance of these results to a wider range of potential clinical trials. For large neonatal trials in the United Kingdom, we demonstrate that the core non-outcome data items identified here are held in the NNRD to a high degree of completeness. For some core non-outcome data items, such as gestational age at birth, we show that the likelihood of missing data in clinical trials utilising the NNRD is small. These results can be used to develop and apply approaches to improve the recording of critical data items with lower completeness in a targeted way; for example, mode of delivery.

Common datasets in other clinical and research areas have been identified using a variety of methods. Doods et al. [62] identified common data groups and elements for feasibility analysis in cardiovascular medicine, diabetes, inflammatory, oncology and neurology through the use of an expert panel, but did not review the literature or include expertise from outside the field. This study identified a wide range of laboratory tests for feasibility studies. Diagnostic test data were not identified in our systematic review of large neonatal trials as commonly reported non-outcome data items, indicating that such data items are not as relevant to the pragmatic neonatal trials that are the focus of this work. Sheehan at al [63]. outline previously developed common data element sets, and some of the challenges inherent in adopting and using such sets. Chari et al. [64] conducted a systematic review of included trials and observational studies to identify common data elements in chronic subdural haematoma studies and, in keeping with our results, identified a core set of commonly reported non-outcome items. The approach that we used was a more limited systematic review of trials published in high-impact journals. This approach was chosen a-priori to focus on data items reported in trials that influence neonatal practice. This was a pragmatic decision and there are limitations to this approach: by limiting our review to general medical journals we may have missed influential trials published in specialty journals, and have not sampled the range of outcomes reported in smaller trials. Furthermore, no approach to date has sought parent or patient views on the importance of different non-outcome data items; this may be important given the different priorities identified by these groups compared to health professionals and researchers [65]. The examples cited here demonstrate the interest in, and potential value of, common sets of non-outcome data items, across different specialties. The development of an established methodological approach, analogous to that developed by the COMET initiative [12] would increase the consistency, robustness and comparability of such endeavours in future.

Our study has focussed on defining the data items usually recorded at baseline or used as explanatory data items in clinical trials. To our best knowledge there have been no previous attempts to identify core non-outcome trial data items such as these. We included the most common data items used in randomisation, which are often selected to conduct pre-specified subgroup analyses, and to adjust for the primary outcome. These items are often overlooked when exploring the impact of data quality in trials, despite the importance of completeness of these items for preserving statistical power and avoiding misinterpretation of results. We did not focus on outcome data items because the methodology to identify these data is well developed and such work is underway in neonatal medicine [16]. A limitation of our study is that data may have been selectively reported thus introducing bias; however, this is lessened as the included journal review protocols are designed to ensure that those items listed in the protocol are presented in the main trial outcomes publication. A further limitation of our study was that some items identified were dichotomous; for example, presence or absence of infection prior to trial enrolment and it was not possible to calculate completeness for such items as absence of the condition is not always actively recorded. Age was found to be a common data item; however, it is calculated using gestational age which is highly complete in the NNRD and, therefore, completeness for age was not calculated. An additional limitation stems from the fact that some data items collected in clinical trials did not directly align with data items in the NNRD; therefore, there may be a loss of information from aggregating several data items into a common data item held by the NNRD to assess data quality. Furthermore, included trials used different approaches to ascertain commonly reported data items; for example, the most commonly reported data item – gestational age – may be derived from maternal reported data, ultrasound measurement or clinical evaluation. Data held within the NNRD are extracted from routine clinical information used to inform clinical care, these clinically relevant data may be more appropriate for pragmatic trials than more granular data items reported in trials. Differences between trials and routinely recorded data sources in how data items are ascertained and synthesised have the potential to introduce biases into clinical trials seeking to use such routinely recorded data. Where such differences are randomly distributed between trial arms, the impact may be limited to lower precision, rather than systematic bias in favour of one trial arm. Further exploration is needed to understand how to accurately assess and synthesise similar data items and to quantify the direction and magnitude of potential biases.

It is important to note that some NNRD data items had between 10 and 30% missing data. The implications of such degrees of missingness depend on the role of the data item in the trial, but are likely to lead to a loss of precision [66]. Baseline variables have a role in pre-specified statistical analyses of outcomes in order that treatment effects can be estimated more precisely. Where the baseline is missing, there are methods which do allow incomplete baseline variables to be included without removing the patients with missing baselines, and to achieve some increase in precision. This is relevant to individually randomised trials, whereas an incomplete baseline may have a greater impact in trials randomising centre clusters when baseline completeness varies by centre. Baseline variables are also used to describe the trial population; for example, to allow readers to judge generalisability, and a high level of baseline completeness may be important for this purpose. Finally, baseline variables are important for subgroup analyses and missing data may limit such analyses. The results presented here will allow the impact that different degrees of missingness have in neonatal trials to be further explored and modelled to better understand which trials are most suitable to use routinely recorded data. The more widespread use of routinely collected data for clinical trials also has the potential to improve the recording of such data [67]. Another limitation is that we did not evaluate the accuracy of common non-outcome data items in the NNRD in this study, although this has recently been undertaken [17]. Completeness and accuracy are key factors in determining the suitability of using routinely recorded clinical data for clinical trials and should be evaluated for all data items deemed critical to any trial seeking to use such data.

The clinical and economic efficiency of using routinely recorded common data items has been demonstrated by trials that have used common registries such as SWEDEHEART [68, 69]. Common data items, as identified here and in core outcome sets [70], can be used to ensure that existing primary data capture systems such as EPR systems and registries capture appropriate data for trials, and in planning such trials. High accuracy and completeness of data are critical for trials; it may, however, not be feasible to evaluate such metrics for all data items within a database or registry – common data items and core outcome sets can be used to target quality assessment of data items most critical to a range of clinical trials. Ongoing data-enabled pilot trials that use routinely recorded data held in the NNRD (15) should provide prospective data regarding the feasibility of such an approach in the neonatal field.

## Conclusion

Neonatal trials in high-impact journals report a common set of non-outcome data items in their primary publications. In the UK, our study indicates that these core non-outcome data can be obtained from the NNRD; the feasibility and efficiency using routinely recorded EPR data such as that held in the NNRD for neonatal clinical trials, rather than collecting these items anew, should be examined. We suggest that when planning primary data collection systems such as EPR systems, registries or clinical databases, consideration is given to fostering a culture of completeness and ensuring that important items are accurately and completely captured.

## Supplementary information

**Additional file 1.** NeoCODE PubMed search strategy.

**Additional file 2: Table S1.** All baseline data items reported by the studies stratified by whether the study recruited preterm or term infants. Data items refer to infant chanracteristics unless otherwise stated. **Table S2.** The most common baseline data items by each identified study. Black indicates that the study presented the data item at baseline. **Table S3.** All data items used as stratifying items during randomisation reported by the studies and by the age of infants included in the studies. **Table S4.** All data items used as confounders to adjust the primary outcome reported by the studies and by the age of infants included in the studies

## Data Availability

The datasets analysed during the current study are available in the National Neonatal Research Database; https://www.imperial.ac.uk/neonatal-data-analysis-unit/neonatal-data/utilising-the-nnrd/

## Abbreviations

caDSR: Cancer Data Standards Registry
EPR: Electronic Patient Records
NDAU: Neonatal Data Analysis Unit
NHS: National Health Service
NNRD: National Neonatal Research Database
PROMIS: Patient Reported Outcomes Measurement Information System

## References

[1] Sinclair JC, Haughton DE, Bracken MB, Horbar JD, Soll RF (2003). Cochrane neonatal systematic reviews: a survey of the evidence for neonatal therapies. Clin Perinatol.

[2] Willhelm C, Girisch W, Gottschling S, Graber S, Wahl H, Meyer S (2013). Systematic Cochrane reviews in neonatology: a critical appraisal. Pediatr Neonatol.

[3] Collier R (2009). Rapidly rising clinical trial costs worry researchers. CMAJ.

[4] Speich B, von Niederhausern B, Schur N, Hemkens LG, Furst T, Bhatnagar N (2018). Systematic review on costs and resource use of randomized clinical trials shows a lack of transparent and comprehensive data. J Clin Epidemiol.

[5] NIHR (2019). NIHR Journals Library.

[6] Le Jeannic A, Quelen C, Alberti C, Durand-Zaleski I, CompaRec I (2014). Comparison of two data collection processes in clinical studies: electronic and paper case report forms. BMC Med Res Methodol.

[7] Lauer MS, D'Agostino RB (2013). The randomized registry trial—the next disruptive technology in clinical research?. N Engl J Med.

[8] Grossmann C, Sanders J, English RA (2013). Large simple trials and knowledge generation in a learning healthcare system. Institute of Medicine.

[9] Eapen ZJ, Lauer MS, Temple RJ (2014). The imperative of overcoming barriers to the conduct of large, simple trials. JAMA.

[10] Ford I, Norrie J (2016). Pragmatic trials. N Engl J Med.

[11] Kopcke F, Trinczek B, Majeed RW, Schreiweis B, Wenk J, Leusch T (2013). Evaluation of data completeness in the electronic health record for the purpose of patient recruitment into clinical trials: a retrospective analysis of element presence. BMC Med Inform Decis Mak.

[12] Williamson PR, Altman DG, Bagley H, Barnes KL, Blazeby JM, Brookes ST (2017). The COMET Handbook: version 1.0. Trials.

[13] Digital N. National Neonatal Data Set NHS Data Dictionary; [Available from: https://www.datadictionary.nhs.uk/data_dictionary/messages/clinical_data_sets/overviews/national_neonatal_data_set_overviews/national_neonatal_data_set_introduction.asp?shownav=1?query=%22national+neonatal%22&rank=100&shownav=1. Accessed 16 Oct 2019.

[14] Gale C, Modi N, Jawad S, Culshaw L, Dorling J, Bowler U (2019). The WHEAT pilot trial-WithHolding Enteral feeds Around packed red cell Transfusion to prevent necrotising enterocolitis in preterm neonates: a multicentre, electronic patient record (EPR), randomised controlled point-of-care pilot trial. BMJ Open.

[15] Reuters T. InCites Journal Citation Reports [Available from: https://jcr.incites.thomsonreuters.com. Accessed 16 Oct 2019.

[16] Webbe J, Brunton G, Ali S, Duffy JMN, Modi N, Gale C (2017). Developing, implementing and disseminating a core outcome set for neonatal medicine. BMJ Paediatrics Open.

[17] Battersby C, Statnikov Y, Santhakumaran S, Gray D, Modi N, Costeloe K (2018). The United Kingdom National Neonatal Research Database: a validation study. PLoS One.

[18] Azzopardi DV, Strohm B, Edwards AD, Dyet L, Halliday HL, Juszczak E (2009). Moderate hypothermia to treat perinatal asphyxial encephalopathy. N Engl J Med.

[19] Azzopardi D, Strohm B, Marlow N, Brocklehurst P, Deierl A, Eddama O (2014). Effects of hypothermia for perinatal asphyxia on childhood outcomes. N Engl J Med.

[20] Ballard RA, Truog WE, Cnaan A, Martin RJ, Ballard PL, Merrill JD (2006). Inhaled nitric oxide in preterm infants undergoing mechanical ventilation. N Engl J Med.

[21] Bassler D, Plavka R, Shinwell ES, Hallman M, Jarreau PH, Carnielli V (2015). Early inhaled budesonide for the prevention of bronchopulmonary dysplasia. N Engl J Med.

[22] Baud O, Maury L, Lebail F, Ramful D, El Moussawi F, Nicaise C (2016). Effect of early low-dose hydrocortisone on survival without bronchopulmonary dysplasia in extremely preterm infants (PREMILOC): a double-blind, placebo-controlled, multicentre, randomised trial. Lancet.

[23] Beardsall K, Vanhaesebrouck S, Ogilvy-Stuart AL, Vanhole C, Palmer CR, van Weissenbruch M (2008). Early insulin therapy in very-low-birth-weight infants. N Engl J Med.

[24] Benjamin DK, Hudak ML, Duara S, Randolph DA, Bidegain M, Mundakel GT (2014). Effect of fluconazole prophylaxis on candidiasis and mortality in premature infants: a randomized clinical trial. JAMA.

[25] Brocklehurst P, Farrell B, King A, Juszczak E, Darlow B, Haque K (2011). Treatment of neonatal sepsis with intravenous immune globulin. N Engl J Med.

[26] Carlo WA, Finer NN, Walsh MC, Rich W, Gantz MG, Laptook AR (2010). Target ranges of oxygen saturation in extremely preterm infants. N Engl J Med.

[27] Carr R, Brocklehurst P, Dore CJ, Modi N (2009). Granulocyte-macrophage colony stimulating factor administered as prophylaxis for reduction of sepsis in extremely preterm, small for gestational age neonates (the PROGRAMS trial): a single-blind, multicentre, randomised controlled trial. Lancet.

[28] Ceelie I, de Wildt SN, van Dijk M, van den Berg MM, van den Bosch GE, Duivenvoorden HJ (2013). Effect of intravenous paracetamol on postoperative morphine requirements in neonates and infants undergoing major noncardiac surgery: a randomized controlled trial. JAMA.

[29] Costeloe K, Hardy P, Juszczak E, Wilks M, Millar MR, Study PPI (2016). *Bifidobacterium breve* BBG-001 in very preterm infants: a randomised controlled phase 3 trial. Lancet.

[30] Davidson AJ, Disma N, de Graaff JC, Withington DE, Dorris L, Bell G (2016). Neurodevelopmental outcome at 2 years of age after general anaesthesia and awake-regional anaesthesia in infancy (GAS): an international multicentre, randomised controlled trial. Lancet.

[31] Fergusson DA, Hebert P, Hogan DL, LeBel L, Rouvinez-Bouali N, Smyth JA (2012). Effect of fresh red blood cell transfusions on clinical outcomes in premature, very low-birth-weight infants The ARIPI randomized trial. JAMA.

[32] Finer NN, Carlo WA, Walsh MC, Rich W, Gantz MG, Laptook AR (2010). Early CPAP versus surfactant in extremely preterm infants. N Engl J Med.

[33] Fivez T, Kerklaan D, Mesotten D, Verbruggen S, Wouters PJ, Vanhorebeek I (2016). Early versus late parenteral nutrition in critically ill children. N Engl J Med.

[34] Gopel W, Kribs A, Ziegler A, Laux R, Hoehn T, Wieg C (2011). Avoidance of mechanical ventilation by surfactant treatment of spontaneously breathing preterm infants (AMV): an open-label, randomised, controlled trial. Lancet.

[35] Harris DL, Weston PJ, Signal M, Chase JG, Harding JE (2013). Dextrose gel for neonatal hypoglycaemia (the Sugar Babies Study): a randomised, double-blind, placebo-controlled trial. Lancet.

[36] Hyttel-Sorensen S., Pellicer A., Alderliesten T., Austin T., van Bel F., Benders M., Claris O., Dempsey E., Franz A. R., Fumagalli M., Gluud C., Grevstad B., Hagmann C., Lemmers P., van Oeveren W., Pichler G., Plomgaard A. M., Riera J., Sanchez L., Winkel P., Wolf M., Greisen G. (2015). Cerebral near infrared spectroscopy oximetry in extremely preterm infants: phase II randomised clinical trial. BMJ.

[37] Kelleher J, Bhat R, Salas AA, Addis D, Mills EC, Mallick H (2013). Oronasopharyngeal suction versus wiping of the mouth and nose at birth: a randomised equivalency trial. Lancet.

[38] Kimberlin DW, Whitley RJ, Wan W, Powell DA, Storch G, Ahmed A (2011). Oral acyclovir suppression and neurodevelopment after neonatal herpes. N Engl J Med.

[39] Kimberlin DW, Jester PM, Sanchez PJ, Ahmed A, Arav-Boger R, Michaels MG (2015). Valganciclovir for symptomatic congenital cytomegalovirus disease. N Engl J Med.

[40] Kirpalani H, Millar D, Lemyre B, Yoder BA, Chiu A, Roberts RS (2013). A trial comparing noninvasive ventilation strategies in preterm infants. N Engl J Med.

[41] Leuchter RHV, Gui L, Poncet A, Hagmann C, Lodygensky GA, Martin E (2014). Association between early administration of high-dose erythropoietin in preterm infants and brain MRI abnormality at term-equivalent age. JAMA.

[42] Makrides M, Gibson RA, McPhee AJ, Collins CT, Davis PG, Doyle LW (2009). Neurodevelopmental outcomes of preterm infants fed high-dose docosahexaenoic acid a randomized controlled trial. JAMA.

[43] Manley BJ, Owen LS, Doyle LW, Andersen CC, Cartwright DW, Pritchard MA (2013). High-flow nasal cannulae in very preterm infants after extubation. N Engl J Med.

[44] Manzoni P, Stolfi I, Pugni L, Decembrino L, Magnani C, Vetrano G (2007). A multicenter, randomized trial of prophylactic fluconazole in preterm neonates. N Engl J Med.

[45] Manzoni P, Rinaldi M, Cattani S, Pugni L, Romeo MG, Messner H (2009). Bovine lactoferrin supplementation for prevention of late-onset sepsis in very low-birth-weight neonates: a randomized trial. JAMA.

[46] Mercier JC, Hummler H, Durrmeyer X, Sanchez-Luna M, Carnielli V, Field D (2010). Inhaled nitric oxide for prevention of bronchopulmonary dysplasia in premature babies (EUNO): a randomised controlled trial. Lancet.

[47] Morley CJ, Davis PG, Doyle LW, Brion LP, Hascoet JM, Carlin JB (2008). Nasal CPAP or intubation at birth for very preterm infants. N Engl J Med.

[48] Morris BH, Oh W, Tyson JE, Stevenson DK, Phelps DL, O’Shea TM (2008). Aggressive vs. conservative phototherapy for infants with extremely low birth weight. N Engl J Med.

[49] Morris RK, Malin GL, Quinlan-Jones E, Middleton LJ, Hemming K, Burke D (2013). Percutaneous vesicoamniotic shunting versus conservative management for fetal lower urinary tract obstruction (PLUTO): a randomised trial. Lancet.

[50] Moss RL, Dimmitt RA, Barnhart DC, Sylvester KG, Brown RL, Powell DM (2006). Laparotomy versus peritoneal drainage for necrotizing enterocolitis and perforation. N Engl J Med.

[51] Natalucci G, Latal B, Koller B, Rugger C, Sick B, Held L (2016). Effect of early prophylactic high-dose recombinant human erythropoietin in very preterm infants on neurodevelopmental outcome at 2 years. A randomized clinical trial. JAMA.

[52] Schmidt B, Anderson PJ, Doyle LW, Dewey D, Grunau RE, Asztalos EV (2012). Survival without disability to age 5 years after neonatal caffeine therapy for apnea of prematurity. JAMA.

[53] Schmidt B, Whyte RK, Asztalos EV, Moddemann D, Poets C, Rabi Y (2013). Effects of targeting higher vs lower arterial oxygen saturations on death or disability in extremely preterm infants: a randomized clinical trial. JAMA..

[54] Shankaran S, Pappas A, McDonald SA, Vohr BR, Hintz SR, Yolton K (2012). Childhood outcomes after hypothermia for neonatal encephalopathy. N Engl J Med.

[55] Shankaran S, Laptook AR, Pappas A, McDonald SA, Das A, Tyson JE (2014). Effect of depth and duration of cooling on deaths in the NICU among neonates with hypoxic ischemic encephalopathy. A randomized clinical trial. JAMA..

[56] Slater R, Cornelissen L, Fabrizi L, Patten D, Yoxen J, Worley A (2010). Oral sucrose as an analgesic drug for procedural pain in newborn infants: a randomised controlled trial. Lancet.

[57] Stenson BJ, Tarnow-Mordi WO, Darlow BA, Simes J, Juszczak E, Askie L (2013). Oxygen saturation and outcomes in preterm infants. N Engl J Med.

[58] Taddio A, Lee C, Yip A, Parvez B, McNamara PJ, Shah V (2006). Intravenous morphine and topical tetracaine for treatment of pain in preterm neonates undergoing central line placement. JAMA.

[59] Tarnow-Mordi W, Stenson B, Kirby A, Juszczak E, Donoghoe M, Deshpande S (2016). Outcomes of two trials of oxygen-saturation targets in preterm infants. N Engl J Med.

[60] Vaucher YE, Peralta-Carcelen M, Finer NN, Carlo WA, Gantz MG, Walsh MC (2012). Neurodevelopmental outcomes in the early CPAP and pulse oximetry trial. N Engl J Med.

[61] Zivanovic S, Peacock J, Alcazar-Paris M, Lo JW, Lunt A, Marlow N (2014). Late outcomes of a randomized trial of high-frequency oscillation in neonates. N Engl J Med.

[62] Doods J, Botteri F, Dugas M, Fritz F, Ehr4Cr WP (2014). A European inventory of common electronic health record data elements for clinical trial feasibility. Trials.

[63] Sheehan J, Hirschfeld S, Foster E, Ghitza U, Goetz K, Karpinski J (2016). Improving the value of clinical research through the use of Common Data Elements. Clin Trials.

[64] Chari A, Hocking KC, Edlmann E, Turner C, Santarius T, Hutchinson PJ (2016). Core outcomes and common data elements in chronic subdural hematoma: a systematic review of the literature focusing on baseline and peri-operative care data elements. J Neurotrauma.

[65] Webbe J, Brunton G, Ali S, Longford N, Modi N, Gale C (2018). Parent, patient and clinician perceptions of outcomes during and following neonatal care: a systematic review of qualitative research. BMJ Paediatr Open.

[66] White IR, Thompson SG (2005). Adjusting for partially missing baseline measurements in randomized trials. Stat Med.

[67] Gale C, Modi N, group Wtd. Neonatal randomised point-of-care trials are feasible and acceptable in the UK: results from two national surveys. Arch Dis Child Fetal Neonatal Ed. 2016;101(1):F86–7. 10.1136/archdischild-2015-308882 PMC4717377 26500239

[68] Frobert O, Lagerqvist B, Olivecrona GK, Omerovic E, Gudnason T, Maeng M (2013). Thrombus aspiration during ST-segment elevation myocardial infarction. N Engl J Med.

[69] Hofmann R, James SK, Jernberg T, Lindahl B, Erlinge D, Witt N (2017). Oxygen therapy in suspected acute myocardial infarction. N Engl J Med.

[70] Webbe J, Sinha I, Gale C (2018). Core outcome sets. Arch Dis Child Educ Pract Ed.

