# Supplementary material for: A systematic review identifying common data items in neonatal trials and assessing their completeness in routinely recorded United Kingdom national neonatal data
Source: Trials. 2019 Dec 16;20:731. doi: 10.1186/s13063-019-3849-7 (PMC6915866; doi:10.1186/s13063-019-3849-7)
Supplement: Supplementary file 1 — Additional file 1. NeoCODE PubMed search strategy. [file 13063_2019_3849_MOESM1_ESM.docx]

**Additional file 1**

NeoCODE Pubmed search strategy

(((neonat*) OR preterm)) AND ((((BMJ.[Journal]) OR JAMA.[Journal]) OR N Engl J Med.[Journal]) OR Lancet.[Journal])

- Limited to Humans
- Limited to Clinical Trials
- Between 2006/01/01 to 2015/12/31
