# Supplementary material for: A systematic review identifying common data items in neonatal trials and assessing their completeness in routinely recorded United Kingdom national neonatal data
Source: Trials. 2019 Dec 16;20:731. doi: 10.1186/s13063-019-3849-7 (PMC6915866; doi:10.1186/s13063-019-3849-7)
Supplement: Supplementary file 2 — Additional file 2: Table S1. All baseline data items reported by the studies stratified by whether the study recruited preterm or term infants. Data items refer to infant chanracteristics unless otherwise stated. Table S2. The most common baseline data items by each identified study. Black indicates that the study presented the data item at baseline. Table S3. All data items used as stratifying items during randomisation reported by the studies and by the age of infants included in the studies. Table S4. All data items used as confounders to adjust the primary outcome reported by the studies and by the age of infants included in the studies [file 13063_2019_3849_MOESM2_ESM.docx]

**Table S1- All baseline data items reported by the studies stratified by whether the study recruited preterm or term infants. Data items refer to infant chanracteristics unless otherwise stated.**

|  | Preterm Studies  (n=29) | | Term Studies  (n=6) | | Mixed Ages Studies  (n=9) | | All Studies  (n=44) | |
| --- | --- | --- | --- | --- | --- | --- | --- | --- |
| Gestational age | 29 | 100% | 4 | 67% | 9 | 100% | 42 | 95% |
| Sex | 29 | 100% | 6 | 100% | 6 | 67% | 41 | 93% |
| Birth weight | 29 | 100% | 5 | 83% | 6 | 67% | 40 | 91% |
| Antenatal steroids | 25 | 86% | 1 | 17% | 1 | 11% | 27 | 61% |
| Multiple birth | 21 | 72% | 1 | 17% | 2 | 22% | 24 | 55% |
| Respiratory Support | 17 | 59% | 3 | 50% | 3 | 33% | 23 | 52% |
| Mode of delivery | 14 | 48% | 2 | 33% | 5 | 56% | 21 | 48% |
| Infection | 15 | 52% | 3 | 50% | 3 | 33% | 21 | 48% |
| Drug treatment | 15 | 52% | 0 | 0% | 5 | 56% | 20 | 45% |
| Mother's Ethnicity | 15 | 52% | 1 | 17% | 3 | 33% | 19 | 43% |
| Apgar 5 min | 14 | 48% | 0 | 0% | 5 | 56% | 19 | 43% |
| Age at randomisation | 11 | 38% | 6 | 100% | 2 | 22% | 19 | 43% |
| Born at study hospital | 13 | 45% | 0 | 0% | 2 | 22% | 15 | 34% |
| Mother age | 6 | 21% | 1 | 17% | 6 | 67% | 13 | 30% |
| SES | 5 | 17% | 0 | 0% | 2 | 22% | 7 | 16% |
| Blood test on the neonatal unit | 3 | 10% | 0 | 0% | 3 | 33% | 6 | 14% |
| Head Circumference | 3 | 10% | 1 | 17% | 1 | 11% | 5 | 11% |
| Apgar Score at 1 Minute | 2 | 7% | 1 | 17% | 2 | 22% | 5 | 11% |
| Family Setup | 3 | 10% | 0 | 0% | 2 | 22% | 5 | 11% |
| Temperature | 3 | 10% | 2 | 33% | 0 | 0% | 5 | 11% |
| Mother Parity | 1 | 3% | 0 | 0% | 3 | 33% | 4 | 9% |
| Apgar Score at 10 Minutes | 0 | 0% | 2 | 33% | 2 | 22% | 4 | 9% |
| Early Feeding Characteristics | 4 | 14% | 0 | 0% | 0 | 0% | 4 | 9% |
| pH | 2 | 7% | 0 | 0% | 2 | 22% | 4 | 9% |
| Intraventricular Haemorrhage | 3 | 10% | 0 | 0% | 1 | 11% | 4 | 9% |
| Umbilical cord blood tests | 3 | 10% | 0 | 0% | 1 | 11% | 4 | 9% |
| Birth Length | 2 | 7% | 0 | 0% | 1 | 11% | 3 | 7% |
| Pre-eclampsia | 2 | 7% | 0 | 0% | 1 | 11% | 3 | 7% |
| Retinopathy | 2 | 7% | 0 | 0% | 1 | 11% | 3 | 7% |
| Smoking during pregnancy | 2 | 7% | 0 | 0% | 1 | 11% | 3 | 7% |
| Patent Ductus Arteriosus | 2 | 7% | 0 | 0% | 1 | 11% | 3 | 7% |
| Umbilical Arterial pH | 2 | 7% | 0 | 0% | 1 | 11% | 3 | 7% |
| Weight | 1 | 3% | 1 | 17% | 1 | 11% | 3 | 7% |
| Necrotising Enterocolitis | 3 | 10% | 0 | 0% | 0 | 0% | 3 | 7% |
| Mother BMI | 0 | 0% | 0 | 0% | 2 | 22% | 2 | 5% |
| Amplitude Integrated Electroencephalography | 0 | 0% | 2 | 33% | 0 | 0% | 2 | 5% |
| Delivery Complications | 0 | 0% | 2 | 33% | 0 | 0% | 2 | 5% |
| Clinical Seizures | 0 | 0% | 2 | 33% | 0 | 0% | 2 | 5% |
| Clinical Risk Index for Babies Score | 2 | 7% | 0 | 0% | 0 | 0% | 2 | 5% |
| Neutropenia | 2 | 7% | 0 | 0% | 0 | 0% | 2 | 5% |
| Hypertension | 1 | 3% | 0 | 0% | 1 | 11% | 2 | 5% |
| Blood glucose | 0 | 0% | 0 | 0% | 2 | 22% | 2 | 5% |
| Score for Neonatal Acute Physiology II Score | 2 | 7% | 0 | 0% | 0 | 0% | 2 | 5% |
| Diabetic Mother | 0 | 0% | 0 | 0% | 2 | 22% | 2 | 5% |
| White cell count | 1 | 3% | 0 | 0% | 1 | 11% | 2 | 5% |
| Bronchopulmonary Dysplasia | 2 | 7% | 0 | 0% | 0 | 0% | 2 | 5% |
| Base Deficit | 0 | 0% | 0 | 0% | 2 | 22% | 2 | 5% |
| Seizure | 0 | 0% | 0 | 0% | 2 | 22% | 2 | 5% |
| Encephalopathy | 0 | 0% | 0 | 0% | 2 | 22% | 2 | 5% |
| Complications in pregnancy | 0 | 0% | 0 | 0% | 2 | 22% | 2 | 5% |
| Intrapartum complications | 0 | 0% | 0 | 0% | 2 | 22% | 2 | 5% |
| Mother in Labour | 1 | 3% | 0 | 0% | 1 | 11% | 2 | 5% |
| Duration of surgery | 0 | 0% | 1 | 17% | 1 | 11% | 2 | 5% |
| Haemoglobin level at NICU | 1 | 3% | 0 | 0% | 1 | 11% | 2 | 5% |
| Partial pressure of carbon dioxide before extubation | 2 | 7% | 0 | 0% | 0 | 0% | 2 | 5% |
| FiO2 | 2 | 7% | 0 | 0% | 0 | 0% | 2 | 5% |
| Periventricular Leukomalacia | 2 | 7% | 0 | 0% | 0 | 0% | 2 | 5% |
| Umbilical cord packed cell volume | 0 | 0% | 1 | 17% | 0 | 0% | 1 | 2% |
| Mother weight | 0 | 0% | 1 | 17% | 0 | 0% | 1 | 2% |
| Mother haemoglobin | 0 | 0% | 1 | 17% | 0 | 0% | 1 | 2% |
| Umbilical Cord Haemoglobin | 1 | 3% | 0 | 0% | 0 | 0% | 1 | 2% |
| Blood Group | 1 | 3% | 0 | 0% | 0 | 0% | 1 | 2% |
| Amniotic Fluid Volume | 0 | 0% | 0 | 0% | 1 | 11% | 1 | 2% |
| Renal Pelvis Dilatation | 0 | 0% | 0 | 0% | 1 | 11% | 1 | 2% |
| Renal Pelvis Severe Hydronephrosis | 0 | 0% | 0 | 0% | 1 | 11% | 1 | 2% |
| Macrocystic Renal Appearance | 0 | 0% | 0 | 0% | 1 | 11% | 1 | 2% |
| Respiratory support through endotracheal tube | 1 | 3% | 0 | 0% | 0 | 0% | 1 | 2% |
| Vitamin A | 1 | 3% | 0 | 0% | 0 | 0% | 1 | 2% |
| Respiratory severity score | 1 | 3% | 0 | 0% | 0 | 0% | 1 | 2% |
| Clinical complications | 1 | 3% | 0 | 0% | 0 | 0% | 1 | 2% |
| Epidural | 0 | 0% | 1 | 17% | 0 | 0% | 1 | 2% |
| Neutrophil count | 1 | 3% | 0 | 0% | 0 | 0% | 1 | 2% |
| Cranial ultrasound abnormality | 1 | 3% | 0 | 0% | 0 | 0% | 1 | 2% |
| Proteinuria | 1 | 3% | 0 | 0% | 0 | 0% | 1 | 2% |
| Surgical procedure | 0 | 0% | 1 | 17% | 0 | 0% | 1 | 2% |
| Surgical stress | 0 | 0% | 1 | 17% | 0 | 0% | 1 | 2% |
| PRISM3 | 0 | 0% | 1 | 17% | 0 | 0% | 1 | 2% |
| PIM2 | 0 | 0% | 1 | 17% | 0 | 0% | 1 | 2% |
| hearing defects | 0 | 0% | 0 | 0% | 1 | 11% | 1 | 2% |
| Language Spoken | 0 | 0% | 0 | 0% | 1 | 11% | 1 | 2% |
| STRONGkids risk | 0 | 0% | 1 | 17% | 0 | 0% | 1 | 2% |
| PELOD score | 0 | 0% | 1 | 17% | 0 | 0% | 1 | 2% |
| Emergency admission | 0 | 0% | 1 | 17% | 0 | 0% | 1 | 2% |
| Diagnostic group | 0 | 0% | 1 | 17% | 0 | 0% | 1 | 2% |
| Condition on admission | 0 | 0% | 1 | 17% | 0 | 0% | 1 | 2% |
| Risk factors for neonatal hypoglycaemia | 0 | 0% | 0 | 0% | 1 | 11% | 1 | 2% |
| Weight change during pregnancy | 0 | 0% | 0 | 0% | 1 | 11% | 1 | 2% |
| Intended method of feeding | 0 | 0% | 0 | 0% | 1 | 11% | 1 | 2% |
| Cause of infection | 1 | 3% | 0 | 0% | 0 | 0% | 1 | 2% |
| Bowel perforation or definite NEC | 1 | 3% | 0 | 0% | 0 | 0% | 1 | 2% |
| Surgery in previous 7 days | 1 | 3% | 0 | 0% | 0 | 0% | 1 | 2% |
| Risk of death | 1 | 3% | 0 | 0% | 0 | 0% | 1 | 2% |
| C-reactive protein | 1 | 3% | 0 | 0% | 0 | 0% | 1 | 2% |
| Duration of membrane rupture | 1 | 3% | 0 | 0% | 0 | 0% | 1 | 2% |
| Source of intravenous immune globulin or placebo | 1 | 3% | 0 | 0% | 0 | 0% | 1 | 2% |
| No prenatal care | 0 | 0% | 0 | 0% | 1 | 11% | 1 | 2% |
| Medical insurance | 0 | 0% | 0 | 0% | 1 | 11% | 1 | 2% |
| Magnesium given during Labour | 0 | 0% | 0 | 0% | 1 | 11% | 1 | 2% |
| Meconium-stained amniotic fluid | 0 | 0% | 0 | 0% | 1 | 11% | 1 | 2% |
| HSV type | 0 | 0% | 0 | 0% | 1 | 11% | 1 | 2% |
| HSV DNA | 0 | 0% | 0 | 0% | 1 | 11% | 1 | 2% |
| Evidence of HSV disease on MRI | 0 | 0% | 0 | 0% | 1 | 11% | 1 | 2% |
| CMV disease | 0 | 0% | 0 | 0% | 1 | 11% | 1 | 2% |
| Microcephaly | 0 | 0% | 0 | 0% | 1 | 11% | 1 | 2% |
| Chorioretinitis | 0 | 0% | 0 | 0% | 1 | 11% | 1 | 2% |
| Neuroimaging results | 0 | 0% | 0 | 0% | 1 | 11% | 1 | 2% |
| BSER of best ear | 0 | 0% | 0 | 0% | 1 | 11% | 1 | 2% |
| Previous preterm births | 1 | 3% | 0 | 0% | 0 | 0% | 1 | 2% |
| Total Parenteral Nutrition | 1 | 3% | 0 | 0% | 0 | 0% | 1 | 2% |
| Umbilical cathether positioned | 1 | 3% | 0 | 0% | 0 | 0% | 1 | 2% |
| Duration of stay in NICU | 1 | 3% | 0 | 0% | 0 | 0% | 1 | 2% |
| Central venous catheter positioned | 1 | 3% | 0 | 0% | 0 | 0% | 1 | 2% |
| Hematocrit | 1 | 3% | 0 | 0% | 0 | 0% | 1 | 2% |
| Positive result on Coombs test | 1 | 3% | 0 | 0% | 0 | 0% | 1 | 2% |
| Bilirubin | 1 | 3% | 0 | 0% | 0 | 0% | 1 | 2% |
| Renal echogenicity | 0 | 0% | 0 | 0% | 1 | 11% | 1 | 2% |
| Bladder wall thickness | 0 | 0% | 0 | 0% | 1 | 11% | 1 | 2% |
| Platelet count | 1 | 3% | 0 | 0% | 0 | 0% | 1 | 2% |
| Retinopathy of Prematurity | 1 | 3% | 0 | 0% | 0 | 0% | 1 | 2% |
| Right heel lanced | 0 | 0% | 1 | 17% | 0 | 0% | 1 | 2% |
| Death of infant in delivery room | 1 | 3% | 0 | 0% | 0 | 0% | 1 | 2% |
| Epinephrine in the delivery room | 1 | 3% | 0 | 0% | 0 | 0% | 1 | 2% |
| Fluid or normally sterile body fluid | 1 | 3% | 0 | 0% | 0 | 0% | 1 | 2% |

**Table S2- The most common baseline data items by each identified study. Black indicates the study presented the data item at baseline**

|  |  | **Most common baseline data items** | | | | | | | | | | | | | |
| --- | --- | --- | --- | --- | --- | --- | --- | --- | --- | --- | --- | --- | --- | --- | --- |
|  | **Author and Year** | **Gestational Age** | **Sex** | **Birth Weight** | **Antenatal Steroids** | **Multiple Births** | **Mode of Delivery** | **Respiratory*** | **Maternal Ethnicity** | **Infection** | **Apgar Score at 5 Minutes** | **Infant Age** | **Drug Treatment** | **Maternal Age** | **Inborn** |
| ***Respiratory*** | |  |  |  |  |  |  |  |  |  |  |  |  |  |  |
|  | Ballard 2006 |  |  |  |  |  |  |  |  |  |  |  |  |  |  |
|  | Bassler 2015 |  |  |  |  |  |  |  |  |  |  |  |  |  |  |
|  | Baud 2016 |  |  |  |  |  |  |  |  |  |  |  |  |  |  |
|  | Carlo 2010 |  |  |  |  |  |  |  |  |  |  |  |  |  |  |
|  | Finer 2010 |  |  |  |  |  |  |  |  |  |  |  |  |  |  |
|  | Gopel 2011 |  |  |  |  |  |  |  |  |  |  |  |  |  |  |
|  | Kelleher 2013 |  |  |  |  |  |  |  |  |  |  |  |  |  |  |
|  | Kirpalani 2013 |  |  |  |  |  |  |  |  |  |  |  |  |  |  |
|  | Manley 2013 |  |  |  |  |  |  |  |  |  |  |  |  |  |  |
|  | Mercier 2010 |  |  |  |  |  |  |  |  |  |  |  |  |  |  |
|  | Morley 2008 |  |  |  |  |  |  |  |  |  |  |  |  |  |  |
|  | Schmidt 2012 |  |  |  |  |  |  |  |  |  |  |  |  |  |  |
|  | Schmidt 2013 |  |  |  |  |  |  |  |  |  |  |  |  |  |  |
|  | Stensen 2013 |  |  |  |  |  |  |  |  |  |  |  |  |  |  |
|  | Tarnow-Mordi 2016 |  |  |  |  |  |  |  |  |  |  |  |  |  |  |
|  | Vaucher 2012 |  |  |  |  |  |  |  |  |  |  |  |  |  |  |
|  | Zivanovic 2014 |  |  |  |  |  |  |  |  |  |  |  |  |  |  |
| ***Infection*** | |  |  |  |  |  |  |  |  |  |  |  |  |  |  |
| Benjamin 2014 | |  |  |  |  |  |  |  |  |  |  |  |  |  |  |
| Brocklehurst 2011 | |  |  |  |  |  |  |  |  |  |  |  |  |  |  |
| Carr 2009 | |  |  |  |  |  |  |  |  |  |  |  |  |  |  |
| Costeloe 2016 | |  |  |  |  |  |  |  |  |  |  |  |  |  |  |
|  | Kimberlin 2011 |  |  |  |  |  |  |  |  |  |  |  |  |  |  |
|  | Kimberlin 2015 |  |  |  |  |  |  |  |  |  |  |  |  |  |  |
|  | Manzoni 2007 |  |  |  |  |  |  |  |  |  |  |  |  |  |  |
|  | Manzoni 2009 |  |  |  |  |  |  |  |  |  |  |  |  |  |  |
| ***Neurological*** | |  |  |  |  |  |  |  |  |  |  |  |  |  |  |
| Azzopardi 2009 | |  |  |  |  |  |  |  |  |  |  |  |  |  |  |
| Azzopardi 2014 | |  |  |  |  |  |  |  |  |  |  |  |  |  |  |
| Hyttel-Sorenson 2015 | |  |  |  |  |  |  |  |  |  |  |  |  |  |  |
| Leuchter 2014 | |  |  |  |  |  |  |  |  |  |  |  |  |  |  |
| Natalucci 2016 | |  |  |  |  |  |  |  |  |  |  |  |  |  |  |
|  | Shankaran 2012 |  |  |  |  |  |  |  |  |  |  |  |  |  |  |
|  | Shankaran 2014 |  |  |  |  |  |  |  |  |  |  |  |  |  |  |
| ***Gastrointestinal*** | |  |  |  |  |  |  |  |  |  |  |  |  |  |  |
| Moss 2006 | |  |  |  |  |  |  |  |  |  |  |  |  |  |  |
| ***Genitourinary*** | |  |  |  |  |  |  |  |  |  |  |  |  |  |  |
| Morris 2013 | |  |  |  |  |  |  |  |  |  |  |  |  |  |  |
| ***Other*** | |  |  |  |  |  |  |  |  |  |  |  |  |  |  |
| Beardsall 2008 | |  |  |  |  |  |  |  |  |  |  |  |  |  |  |
| Ceelie 2013 | |  |  |  |  |  |  |  |  |  |  |  |  |  |  |
| Davidson 2016 | |  |  |  |  |  |  |  |  |  |  |  |  |  |  |
| Fergusson 2012 | |  |  |  |  |  |  |  |  |  |  |  |  |  |  |
| Fivez 2016 | |  |  |  |  |  |  |  |  |  |  |  |  |  |  |
| Harris 2013 | |  |  |  |  |  |  |  |  |  |  |  |  |  |  |
| Makrides 2009 | |  |  |  |  |  |  |  |  |  |  |  |  |  |  |
| Morris 2008 | |  |  |  |  |  |  |  |  |  |  |  |  |  |  |
| Slater 2010 | |  |  |  |  |  |  |  |  |  |  |  |  |  |  |
| Taddio 2006 | |  |  |  |  |  |  |  |  |  |  |  |  |  |  |

***** Respiratory refers to respiratory support required prior to trial entry

**Table S3-** **All data items used as stratifying items during randomisation reported by the studies and by the age of infants included in the studies**

| Stratification Items | Preterm Studies  (n=29) | Term Studies  (n=6) | Mixed Ages Studies  (n=9) | All Studies  (n=44) |
| --- | --- | --- | --- | --- |
| Centre | 22 (76%) | 1 (17%) | 2 (22%) | 25 (57%) |
| Gestational age | 13 (45%) | 1 (17%) | 3 (33%) | 17 (39%) |
| Birth weight | 7 (24%) | 0 (0%) | 1 (11%) | 8 (18%) |
| Sex | 4 (14%) | 0 (0%) | 0 (0%) | 4 (9%) |
| Multiple birth | 3 (10%) | 0 (0%) | 0 (0%) | 3 (7%) |
| Inborn | 2 (7%) | 0 (0%) | 0 (0%) | 2 (5%) |
| Infant Age | 0 (0%) | 2 (33%) | 0 (0%) | 2 (5%) |
| Abnormality on amplitude-integrated electroencephalography | 0 (0%) | 1 (17%) | 0 (0%) | 1 (2%) |
| Sibling Enrolment Status | 1 (3%) | 0 (0%) | 0 (0%) | 1 (2%) |
| Randomisation Time from Birth | 1 (3%) | 0 (0%) | 0 (0%) | 1 (2%) |
| Diagnosis on Admission | 0 (0%) | 1 (17%) | 0 (0%) | 1 (2%) |
| Maternal Diabetes | 0 (0%) | 0 (0%) | 1 (11%) | 1 (2%) |
| Prior-Intubation Status | 1 (3%) | 0 (0%) | 0 (0%) | 1 (2%) |
| Age of Mother | 0 (0%) | 0 (0%) | 1 (11%) | 1 (2%) |
| Volume of Amniotic Fluid | 0 (0%) | 0 (0%) | 1 (11%) | 1 (2%) |
| Encephalopathy | 0 (0%) | 0 (0%) | 1 (11%) | 1 (2%) |

**Table S4 - All data items used as confounders to adjust the primary outcome reported by the studies and by the age of infants included in the studies**

| Primary Outcome Confounders | Preterm Studies  (n=29) | Term Studies  (n=6) | Mixed Ages Studies  (n=9) | All Studies  (n=44) |
| --- | --- | --- | --- | --- |
| Gestational Age | 17 (59%) | 1 (17%) | 1 (11%) | 19 (43%) |
| Centre | 10 (34%) | 1 (17%) | 2 (22%) | 13 (30%) |
| Birth Weight | 9 (31%) | 0 (0%) | 1 (11%) | 10 (23%) |
| Sex | 7 (24%) | 1 (17%) | 0 (0%) | 8 (18%) |
| Multiple Births | 7 (24%) | 0 (0%) | 0 (0%) | 7 (16%) |
| Ventilation | 5 (17%) | 0 (0%) | 0 (0%) | 5 (11%) |
| Steroids | 5 (17%) | 0 (0%) | 0 (0%) | 5 (11%) |
| Maternal Education | 3 (10%) | 1 (17%) | 0 (0%) | 4 (9%) |
| Abnormality on Amplitude-Integrated Electroencephalography | 0 (0%) | 1 (17%) | 2 (22%) | 3 (7%) |
| Severity of Illness | 1 (3%) | 1 (17%) | 0 (0%) | 2 (5%) |
| Nutrition | 1 (3%) | 1 (17%) | 0 (0%) | 2 (5%) |
| Infant Age | 0 (0%) | 2 (33%) | 0 (0%) | 2 (5%) |
| Caffeine Use | 1 (3%) | 0 (0%) | 0 (0%) | 1 (2%) |
| Time of Randomisation | 1 (3%) | 0 (0%) | 0 (0%) | 1 (2%) |
| Diagnostic Group | 0 (0%) | 1 (17%) | 0 (0%) | 1 (2%) |
| Maternal Diabetes | 0 (0%) | 0 (0%) | 1 (11%) | 1 (2%) |
| Central Nervous System Involvement | 0 (0%) | 0 (0%) | 1 (11%) | 1 (2%) |
| Risk Factors Possibly Associated with Invasive Fungal Infection | 1 (3%) | 0 (0%) | 0 (0%) | 1 (2%) |
| Use of H2 Blockers | 1 (3%) | 0 (0%) | 0 (0%) | 1 (2%) |
| Daily Milk Intake | 1 (3%) | 0 (0%) | 0 (0%) | 1 (2%) |
| Pneumatosis | 1 (3%) | 0 (0%) | 0 (0%) | 1 (2%) |
| Platelet Count | 1 (3%) | 0 (0%) | 0 (0%) | 1 (2%) |
| Familial Clustering | 1 (3%) | 0 (0%) | 0 (0%) | 1 (2%) |
| Mode of Delivery | 1 (3%) | 0 (0%) | 0 (0%) | 1 (2%) |
